# Examining the utility of near infrared light as pre-exposure therapy to mitigate temporary noise-induced hearing loss in humans

**DOI:** 10.3389/fneur.2024.1366239

**Published:** 2024-04-22

**Authors:** Erin Williams, Kayla Minesinger, Hilary Gallagher, J. R. Stefanson, Nathaniel Bridges, Natalie Jackson, Valerie Stark, Jennifer Coto, Suhrud Rajguru, Kurt Yankaskas, Rick Rogers, Michael E. Hoffer

**Affiliations:** ^1^Department of Otolaryngology, University of Miami Miller School of Medicine, Miami, FL, United States; ^2^Department of Neurological Surgery, University of Miami Miller School of Medicine, Miami, FL, United States; ^3^Department of Biomedical Engineering, University of Miami, Coral Gables, FL, United States; ^4^Air Force Research Laboratory, 711th Human Performance Wing, Airman Systems Directorate, Dayton, OH, United States; ^5^U.S. Army Aeromedical Research Laboratory, Fort Rucker, AL, United States; ^6^Oak Ridge Institute for Science and Education, Oak Ridge, TN, United States; ^7^University of Miami Miller School of Medicine, Miami, FL, United States; ^8^BioInnovations Institute, Natick, MA, United States

**Keywords:** near-infrared light, noise-induced hearing loss, temporary threshold shift, auditory therapeutics, auditory health

## Abstract

**Introduction:**

This study sought to determine the effect of Occupational Safety and Health Administration (OSHA) compliant noise on auditory health and assess whether pre-noise near infrared (NIR) light therapy can mitigate the effects of noise exposure.

**Methods:**

Over four visits, participants (*n* = 30, NCT#: 03834714) with normal hearing completed baseline hearing health assessments followed by exposure to open ear, continuous pink noise at 94 dBA for 15 min. Immediately thereafter, post-noise hearing tests at 3000, 4000, and 6000 Hz and distortion product otoacoustic emissions (DPOAEs) were conducted along with the Modified Rhyme Test (MRT), Masking Level Difference Test (MLD), and Fixed Level Frequency Tests (FLFT) [collectively referred to as the Central and Peripheral Auditory Test Battery (CPATB)] to acquire baseline noise sensitivity profiles. Participants were then randomized to either Active or Sham NIR light therapy for 30 min binaurally to conclude Visit 1. Visit 2 (≥24 and ≤ 48 h from Visit 1) began with an additional 30-min session of Active NIR light therapy or Sham followed by repeat CPATB testing and noise exposure. Post-noise testing was again conducted immediately after noise exposure to assess the effect of NIR light therapy. The remaining visits were conducted following ≥2 weeks of noise rest in a cross-over design (i.e., those who had received Active NIR light therapy in Visits 1 and 2 received Sham therapy in Visits 3 and 4).

**Results:**

Recovery hearing tests and DPOAEs were completed at the end of each visit. Participants experienced temporary threshold shifts (TTS) immediately following noise exposure, with a mean shift of 6.79 dB HL (±6.25), 10.61 dB HL (±6.89), and 7.30 dB HL (±7.25) at 3000, 4000, and 6000 Hz, respectively, though all thresholds returned to baseline at 3000, 4000, and 6000 Hz within 75 min of noise exposure. Paradoxically, Active NIR light therapy threshold shifts were statistically higher than Sham therapy at 3000 Hz (*p* = 0.04), but no other differences were observed at the other frequencies tested. An age sub-analysis demonstrated that TTS among younger adults were generally larger in the Sham therapy group versus Active therapy, though this was not statistically different. There were no differences in CPATB test results across Active or Sham groups. Finally, we observed no changes in auditory function or central processing following noise exposure, suggestive of healthy and resilient inner ears.

**Conclusion:**

In this study, locally administered NIR prior to noise exposure did not induce a significant protective effect in mitigating noise-induced TTS. Further exploration is needed to implement effective dosage and administration for this promising otoprotective therapy.

## Introduction

Noise induced hearing loss (NIHL) is a type of sensorineural hearing loss (SNHL) that occurs due to overexposure of hazardous levels of noise and is completely preventable. It is known that certain exposures to harmful noise results in temporary threshold shifts (TTS) which recover within 48 h (1, 2). However, the trajectory of recovery and long-term effects on hearing are unpredictable and variable. Hypothetical mechanisms of damage include hair cell loss, loss of synaptic connections between hair cells (HCs) and fibers, and a subsequent decrease in neuronal conduction velocity (3–5). Given the preventable nature of NIHL and its established pathophysiology, it is essential to identify and develop therapeutic interventions to ameliorate the consequences of this condition.

Near infrared (NIR) light therapy has been studied *in vitro* and has been shown to reduce oxidative stress caused by both reactive oxygen species (ROS) and reactive nitrogen species (RNS), overactivity of the mitochondria, and the presence of pro-inflammatory cytokines in injured cochlea (6). In animal studies, NIR treatment has successfully protected vital cochlear structures including ribbon synapses and HCs, improving functional recovery of hearing thresholds and therefore lessening TTS-induced sequelae (7, 8). NIR therapy has also been briefly investigated in humans for other hearing disorders, such as tinnitus (9–12). Though most investigations focus on reducing symptom severity, these findings support a promising therapeutic avenue to mitigate progressive cochlear damage.

Presently, the characterization of TTS in humans and the use of NIR to reduce the negative effects of noise exposure have been poorly described in humans. The main limitation in implementing TTS noise protocols in clinical trials is the safety and efficacy of administering noise to humans (i.e., ensuring that the TTS is sufficiently brief and that it does not result in permanent threshold changes or changes in auditory processing). Further, there are no standardized practices for inducing temporary NIHL in the clinical-translational pipeline. Moreover, to our knowledge, there are no known studies investigating the use of therapeutic pre-noise NIR to treat noise-induced effects in humans. Given the lack of characterization of NIR light therapy in humans for NIHL applications, our administration parameters and timepoints were informed by prior literature and were executed within the constraints of our prototype device. Recent studies employing optical coherence tomography (OCT) have successfully achieved non-invasive imaging of the cochlea by using light at the 850 nm wavelength. This technique necessitates the reflection of light to render the cochlea visible, where first the light must traverse the tympanic membrane and subsequently air, the former of which may impede the returning signal. The ability of 850 nm light to reach the cochlea suggests that NIR light, with its similar properties is also likely effectively reach and image the target tissue, confirming that the parameters we chose for our NIR application were methodically sound. In all, the parameters chosen for this NIR application were methodically sound and physically and mechanically feasible given the limitations of an earbud-based design.

In a TTS model where auditory changes are thought to be metabolically driven (13), we hypothesized that upregulation of protective metabolic activity would prophylactically mitigate noise-induced changes. Mechanistically, NIR induces cytochrome C oxidase in the mitochondria to become oxidized, initiating proton transport and significantly upregulating ATP (7, 14). This increase in ATP reduces cell apoptosis and harmful gene expression that would otherwise result in the activation of a series of cell death pathways. Ultimately, this reduces neuroinflammation, downregulates expression of damaging proteins and pro-apoptotic factors, and decreases harmful free radicals including reactive oxygen species and nitric oxidate (6, 7). For NIHL specifically, NIR therapy has been shown to preserve vital cochlear structures and even protect hair cell synaptopathy in preclinical studies (7, 8). Based on the above, we hypothesized that administration of NIR light therapy before acoustic trauma could provide a potential otoprotective mechanism for the upregulation of protective metabolic activity prior to noise, preemptively downregulating or preventing harmful cascades induced by noise (e.g., apoptotic-related hair cell death and accumulation of undesired radicals).

Overall, the aim of this work was to determine the effect of OSHA-compliant, TTS-inducing noise exposures on human auditory health, and to assess whether pre-noise NIR light therapy can mitigate the effects of these noise exposures (i.e., temporary changes in audiometric measurements immediately following noise exposure).

## Methods

The Institutional Review Board (IRB) at the University of Miami Miller School of Medicine granted permission for the conduction of this research (UMiami IRB #20181214; USAARL/AFRL IRB# FWR20180039H). All participants (*n* = 30) signed written informed consent. Males and females between 18 and 45 years of age were recruited. Inclusion criteria for subjects included ≤25 dB HL qualifying (baseline) thresholds for both ears across all frequencies tested from 500 Hz to 6000 Hz. Additionally, subjects were assessed for normal hearing health as demonstrated by normal otoscopic exams, normal distortion product otoacoustic emissions (DPOAEs), and normal middle ear function as determined via tympanometry. Exclusion criteria included screening hearing test failure, pregnant females, adults unable to provide consent, and/or history of significant ear surgery. Subjects were recruited at the University of Miami (UMiami), United States Army Aeromedical Research Laboratory (USAARL), and the Air Force Research Laboratory (AFRL).

### Hearing evaluation and CAP assessments

All hearing and auditory processing assessments were conducted with the Creare, LLC (Hanover, NH) Wireless Automated Hearing Test System (WAHTS) (15, 16), a boothless audiometer controlled with a smartphone or tablet. The WAHTS internal electronics control the sound pressure levels delivered to the ear, and administers a variety of hearing test algorithms, including Békésy-like audiometry, the FLFT, the MLD test, and the MRT. The WAHTS was controlled with TabSINT, a software platform designed for tablet-based distributed studies of hearing for user interface and data handling (17). TabSINT provides the user interface for audiometry (push button) as well as speech-in-noise or central auditory processing tests. Data was then exported into R Studio for analysis.

### Otoacoustic emissions and tympanogram

DPOAEs and tympanograms were collected and assessed using the Titan V2 with DPOAE (Interacoustics, [Assens, Denmark]). Initial tympanograms as detailed above were used to ensure healthy middle ear function. DPOAEs were evoked by two pure tones (f1 and f2) with a primary tone frequency ratio (f2/f1) of 1.21 (2f1-f2). The sound intensity levels of the primary tones were 65 dB SPL (L1) and 55 dB SPL (L2). The minimum signal-to-noise ratio (SNR), or the difference between OAE amplitude and the noise level measured at the corresponding frequency in dB SPL, was set at ≥6 for 0.5, 1, 2, 3, 4, 6, and 8 kHz (18). Results were reported as “Pass” or “Refer” for convenience. Additional DPOAEs were collected as part of the CPATB administered throughout the experimental timeline.

### Modified rhyme test

The MRT was delivered (binaurally) as a multiple-choice speech-intelligibility test consisting of 46 monosyllabic words in 2 lists (92 words total). Following 5 training trials, each list was presented as 46 ensembles of 6 related words that all share a core vowel and either start or end with the same consonantal phoneme (e.g., tent, bent, went) at varying levels of intensity (70, 78 dB SPL) and SNR (−4; +4). Participants identified which of the 6 words in the ensemble was transmitted by selecting the word on the tablet. In these evaluations, the 92-word battery was delivered with the same carrier phase, “You will mark [MRT stimulus], please.” Results were reported as percent correct (19).

### Masking level difference test (MLD)

The MLD was conducted to assess the sensitivity of the auditory system to differences in time and signal amplitude and/or noise. In this version of the MLD test, 33 noise segment trials were delivered binaurally at 65–68 dB SPL root mean square (RMS) in three different conditions by wav files: SoNo (signal and homophasic noise in both ears), SπNo (antiphase signal in 1 ear and homophasic noise in both ears), and No Noise (control). Participants were instructed to press a button on the tablet if they were able to perceive the tone. The test variables of interest include level and phase of the tones compared to the absent stimuli (without tone) acting as a foil. The number of correct identifications for the SoNo and SπNo conditions given by the subject were then translated to a threshold value and the difference was computed. More specfically, MLD was determined as the difference between the signal-to-noise ratio (SNR) for the SoNo and SπNo conditions (20).

### Fixed-level frequency test

The FLFT was administered at 80 dB SPL with the WAHTS system. Subjects used a audiometry protocol tracking method to determine the highest audible frequency threshold, ranging from 500 Hz to 20 kHz (21). The pulsed pure tones began at 8 kHz and gradually increased until the subject indicates they can no longer hear the stimuli and in turn, the frequency gradually decreased. This reversal process continued several times with differing octave step so that the FLFT values were computed as an average frequency over six reversal bands.

### Noise protocol and NIR intervention

#### UMiami and USAARL noise exposure

**Figure 1:**
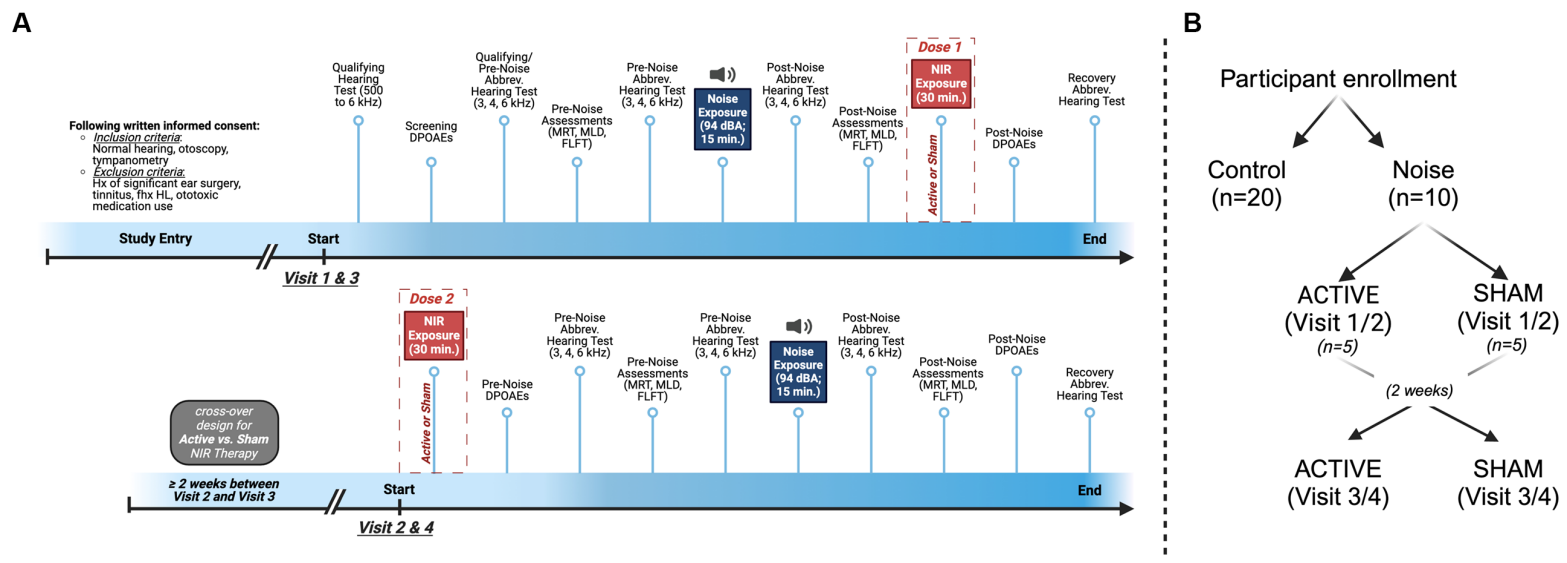
Design and timeline for the experimental group. **(A)** In this cross-over study, visits 1 and 3 began with qualifying hearing test at 3000, 4000, and 6000 Hz followed by the Central and Peripheral Auditory Processing Test Battery (CPATB), which is comprised of the Modified Rhyme Test (MRT), Masking Level Difference Test (MLD), and Fixed Level Frequency Test (FLFT), and Distortion Product Otoacoustic Emissions (DPOAEs). A second pre-noise hearing test was administered prior to noise exposure to ensure individual test–retest reliability. Pink noise was administered at 94 dBA for 15 min, followed by post-noise hearing tests, the CPATB, and 30 min of binaural Active or Sham NIR therapy. Visits concluded with post-therapy DPOAEs and recovery hearing tests (total of 60 min post-noise, including NIR). Visits 2 and 4 began with 30 min of binaural Active or Sham NIR therapy, followed by pre-noise DPOAEs, pre-noise hearing tests, and the CPATB prior to pink noise exposure at 94 dBA for 15 min. Following noise exposure, participants again were administered post-noise hearing tests and the CPATB. Lastly, post-noise DPOAEs and recovery hearing tests were conducted. **(B)** Group breakdown and cross-over design visualization.

Noise was delivered at the ear level of seated participants with mounted speakers placed approximately 5 ± 2 feet away (1x Electro-Voice ZLX 15′ and 3x Electro-Voice T251 [*Burnsville, MN*]). To ensure equal energy level per octave of frequency, pink noise was presented (decay of 3 dB per octave) for 15 min at 94 dBA, as measured by a sound level meter (Figure 1). The noise parameters selected for this study are previously described (22) and were well below the maximum allowable noise exposures and time-weighted average at 94 dBA for 15 min, as determined by the guidelines provided by the National Institute for Occupational Safety and Health (NIOSH) (23) and the Occupational Safety and Health Administration (OSHA).

#### Near-infrared light administration

NIR light was administered for 30 min with a custom headset developed by White Anvil Innovations, LLC (Natick, MA). The engineering prototype was designed with local inner-ear delivery facilitated through ear buds with three size options for insertion into large, medium, and small size ear canals. Each earbud emitted 850 nm light at 58 mW/cm2 at 0.5 cm distance from the tympanic membrane, with an energy density of 3.3 J/cm2. Pulsed light was delivered at 20 Hz for three illumination duty cycles up to three minutes per cycle with up to 45 s delay between the first and second duty cycles. In order to ensure that this prototype did not exceed its thermal operational limits, the designated therapeutic dose of NIR was divided into two subsequent sessions (Visits 1 and 2 or Visits 3 to 4) as described below. No participant experienced untoward or harmful effects from the NIR light therapy device.

### Experimental design

Following written informed consent at Visit 1, ear-specific (i.e., left versus right) pre-noise Bekesy auditory threshold measurements and DPOAEs were collected from subjects to determine qualifying (baseline) auditory responses (Figure 1). Additional pre-noise auditory assessments included the MRT, MLD, FLFT, and an abbreviated hearing test at frequencies of 3, 4, and 6 kHz. All the aforementioned auditory tests (with test frequencies indicated) will henceforth be referred to as the Central and Peripheral Auditory Test Battery (CPATB) (Figure 1B). Following the above, randomized participants with normal hearing (<25 dB hearing level) were exposed to open ear, continuous pink noise at 94 dBA for 15 min. Within 15 min following noise exposure, subjects who reached threshold changes of ≤20 and ≥ 10 dB HL post-noise were permitted to continue participating in the study (*n* = 10) to reduce group variability and to account for homogeneous hearing profiles/sensitivity to noise in our cohort. Those who were excluded following the initial noise exposure were not included in this analysis. This pre-determined identification of outliers established a cohort in which we would be most likely to observe auditory changes related to the NIR treatment rather than other confounding factors. Further, this criteria filtered for participants with measurable TTS so that the effect of NIR would also be observable. Control participants (*n* = 20) sat in silence for 15 min to simulate experimental group test conditions and proceeded to complete the CPATB as described above.

In this cross-over study, participants were randomized to receive Active or Sham NIR therapy at Visit 1. Visit 1 noise was delivered as described above. Immediately following noise, the post-noise CPATB was conducted, followed by the first dose of NIR (or sham) which was delivered binaurally for a duration of 30 min. Both the primary study team administrator and the participant were blinded to the randomization condition. Visit 1 concluded with recovery DPOAEs and a recovery hearing test.

Twenty-four to 48 h following Visit 1, participants returned for Visit 2, where they received pre-noise (30 min binaurally) Active or Sham NIR therapy. This pre-noise treatment (i.e., the consecutive dosages of NIR light therapy given at the end of Visit 1/Visit 3 and at the beginning of Visit 2/Visit 4) was considered the therapeutic dose/intervention for this study. Again, CPATB were collected to obtain pre-noise values before the second session of noise exposure commenced. The second round of the pink noise protocol was delivered and then post-noise CPATB were collected immediately after. Recovery hearing tests and DPOAEs were administered before concluding Visit 2. All post-noise testing including the NIR light therapy session, regardless of group, was conducted within 60 min following noise exposure (Supplementary Table S1).

Two weeks or more after Visits 1 and 2, Visits 3 and 4 were conducted with identical endpoints, apart from the therapy condition where participants received the opposite device (i.e., if participants received Active NIR during Visit 1 & 2, they received Sham NIR during Visit 3 & 4).

### Statistical analysis

No data, including outliers, were removed prior to analysis. Parametric or non-parametric tests were conducted based on the distribution of each group examined, to include threshold shift in active versus sham groups and intra-ear differences. For normally distributed data, parametric tests were used, including paired t-tests and ANOVAs. For non-normally distributed data, non-parametric tests were used, including the Mann–Whitney U test or Kruskal-Wallis test. Adjusted *p*-values using Bonferroni correction was implemented for all statistical analyses to account for the increased risk of Type I error associated with multiple comparisons.

We employed two additional models; (1) a linear mixed effects model to explore the effect of frequency, condition, and their interaction on TTS magnitude and (2) simple linear regression in order to quantify the strength and direction of associations between the demographic covariate/predictor of age and TTS magnitude, our outcome of interest. It should be noted that further regression analyses with additional predictors were deemed unnecessary due to the clear influence of these predictors as is discernible from the study design and cohort characteristics described below. Their impact on the outcome variable was apparent and consistent with the established theoretical framework and the design of our experiment, thus obviating the need for additional computational validation. Based on the statistical model output and a close examination of our sample cohort in this pilot study, an exploratory analysis investigating potential age-related changes was performed assessing young (18 to 28 years; *n* = 7) and middle-aged (32 to 45 years; *n* = 3) adults. Age groups were stratified based on the median age of the cohort. All analyses were performed with the level of significance set at 0.05. All analyses were conducted in R Studio.

## Results

### Cohort characteristics

Overall, 23 male and 7 female participants were enrolled (*n* = 30). The experimental group (i.e., those who received noise exposure) consisted of *n* = 10 participants (9 male and 1 female) with a mean age of 28.17 (SD ± 7.33) years old. We also enrolled *n* = 20 control participants (14 male and 6 female) with a mean age of 27.6 (SD ± 7.34). Qualifying (baseline) auditory thresholds are reflected in Table 1. Although baseline values were variable, no significant differences were determined between groups. Further, audiometric threshold values have been known to vary up to 10 dB HL during repeat testing (24).

**Table 1:** Qualifying (baseline) auditory thresholds for experimental and control groups.

	3000 Hz [Mean (SD)] (dB HL)		4000 Hz [Mean (SD)] (dB HL)		6000 Hz [Mean (SD)] (dB HL)	
	Left ear	Right ear	Left ear	Right ear	Left ear	Right ear
Experimental group	−10.03 (5.50)	−10.27 (4.21)	−9.55 (6.34)	−11.52 (5.68)	0.39 (5.37)	−0.06 (4.96)
Control group	−0.68 (4.89)	2.96 (8.25)	1.89 (5.72)	0.02 (6.78)	17.81 (5.90)	16.28 (8.15)

Prior to pink noise exposure, all groups underwent audiometric testing to determine qualifying (baseline) thresholds (dB HL). The table above summarizes by ear the average threshold across frequencies 3000, 4000, and 6000 Hz for the experimental (noise) group (*n* = 10) and control group (*n* = 20).

### Open ear pink noise exposure caused temporary threshold shifts

**Figure 2:**
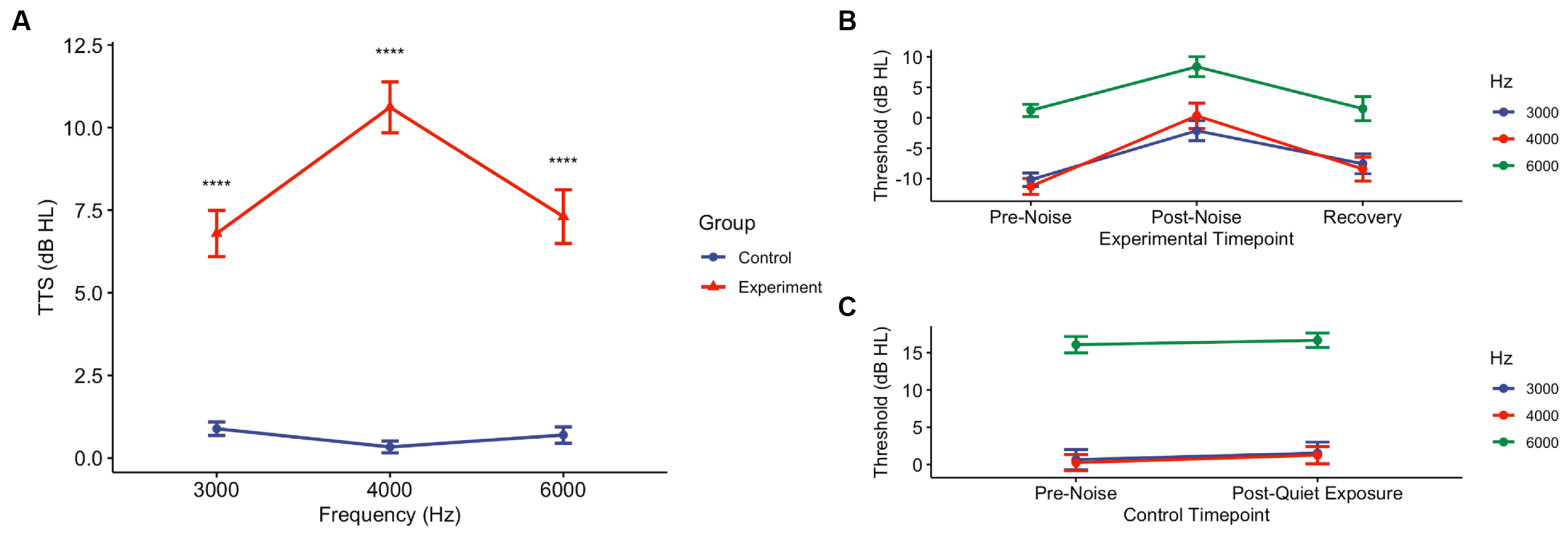
Pink noise exposure at 94 dBA for 15 min induces TTS in the experimental group, though they return to pre-noise levels. **(A)** Post-noise hearing tests collected immediately following exposure period (or no noise) were conducted in experimental and control groups. Threshold shifts were determined using pre-noise thresholds. At all frequencies tested – 3000, 4000, and 6000 Hz – there was a statistical difference between the control and experimental groups (*p* < 0.0001). **(B)** In the experimental (*n* = 10) group, at the conclusion of the post-noise CPATB (and Active/Sham therapy as appropriate), recovery audiometric measurements were taken to ensure that thresholds reverted back to pre-noise levels. All thresholds returned to pre-noise levels (± 5 dB HL). **(C)** Similarly, the control (no noise) group (*n* = 20) underwent audiometric testing following their 15-min quiet period. There were no changes (± 5 dB HL) in pre-noise and post-quiet exposure thresholds at any of the frequencies tested.

After pink noise exposure, regardless of Active or Sham device, all noise-exposed subjects demonstrated temporary threshold shifts at all frequencies tested (Figures 2A,B; Table 2). A Wilcoxon rank sum test comparing the control group to the experimental group demonstrated a mean TTS of 6.79 dB HL (SD ± 6.25) at 3000 Hz (W = 2548.5, *p* < 0.0001), 10.61 dB HL (SD ± 6.89) at 4000 Hz (W = 750, *p* < 0.0001), and 7.30 dB HL (SD ± 7.25) at 6000 Hz (W = 2,541, *p* < 0.0001). We also observed significant pair-wise differences across several frequencies determined by Dunn Kruskal-Wallis multiple comparison tests with Bonferroni correction, specifically between 3000 and 4000 Hz (z = −3.12, *p* = 0.005) and 4000 and 6000 Hz (z = 3.14, *p* = 0.005) in the experimental group. The grand mean TTS (i.e., regardless of threshold tested) was 9.01 dB HL (SD ± 6.29) in the experimental group, while the control group showed a mean TTS of 0.81 dB HL (SD ± 2.52). All experimental participants’ post-noise audiograms returned to within 5 dB HL of their pre-noise thresholds at 3000, 4000, and 6000 Hz within 75 min of noise exposure, while control participants exhibited no threshold shift (Figure 2C). All enrolled participants received a “Pass” result during post-noise DPOAEs, none required referral or further evaluation.

**Table 2:** Mean TTS results for active versus sham therapy groups by condition and ear tested.

Condition	Frequency tested					
	3000 Hz [Mean (SD)]		4000 Hz [Mean (SD)]		6000 Hz [Mean (SD)]	
	Left ear	Right ear	Left ear	Right ear	Left ear	Right ear
Experimental Active NIR therapy TTS	9.07 (6.26)	7.07 (5.85)	11.87 (6.73)	11.24 (8.34)	5.62 (4.86)	8.76 (4.40)
Sham NIR therapy TTS	5.04 (5.26)	3.82 (4.00)	5.04 (5.26)	11.60 (7.25)	4.89 (7.84)	9.77 (6.29)
Control Active NIR therapy TTS	0.25 (2.59)	1.38 (3.05)	1.06 (2.18)	0.94 (2.47)	0.04 (2.58)	1.13 (2.32)
Sham NIR therapy TTS	0.93 (2.78)	1.35 (2.64)	−0.17 (2.04)	0.32 (2.74)	0.07 (3.52)	0.87 (2.33)

After receiving active versus Sham NIR therapy (at either Visit 2 or Visit 4) prior to noise, post-noise audiometric testing was conducted to collect threshold information at 3000, 4000, and 6000 Hz across both ears. No significant difference was found between groups for either ear.

Additionally, after performing an exploratory analysis based on age, it was found that young adults (18 to 28 years; *n* = 7) and middle-aged adults (32 to 45 years; *n* = 3) responded similarly to the experimental noise exposure. Using Welch’s Two Sample t-test in both groups, no ear-specific relationship was found with respect to TTS at any of the frequencies tested (young [t = −0.41, df = 22, *p = 0.68*], middle-aged [t = 1.61, df = 4, *p =* 0.18]). However, pair-wise analysis demonstrated a significant difference between middle-aged and young groups at 3000 and 4000 Hz in which middle-aged subjects exhibited elevated thresholds (3000 Hz: *t* = −3.14, df = 52, *p* = 0.002; 4000 Hz: *t* = 2.83, df = 69, *p* = 0.006).

Control participants had no meaningful change in thresholds at 3000, 4000, or 6000 Hz (Table 2).

### Near-infrared light did not significantly reduce threshold shifts following pink noise exposure

A linear mixed effects model (LME) was fitted to model the effect of NIR therapy on TTS using frequency (3, 4, and 6 kHz) and condition (Active, Sham), as well as their interaction. Neither the main effects of Hz (β = −7.06e-05, t(86.20) = −0.12, *p* = 0.91) nor Condition (β = −4.95, t(84.90) = −1.29, *p* = 0.20) were statistically significant. Similarly, the interaction between Hz and Condition was not significant (β = 5.72e-04, t(84.90)= = 0.65, *p* = 0.52).

**Figure 3:**
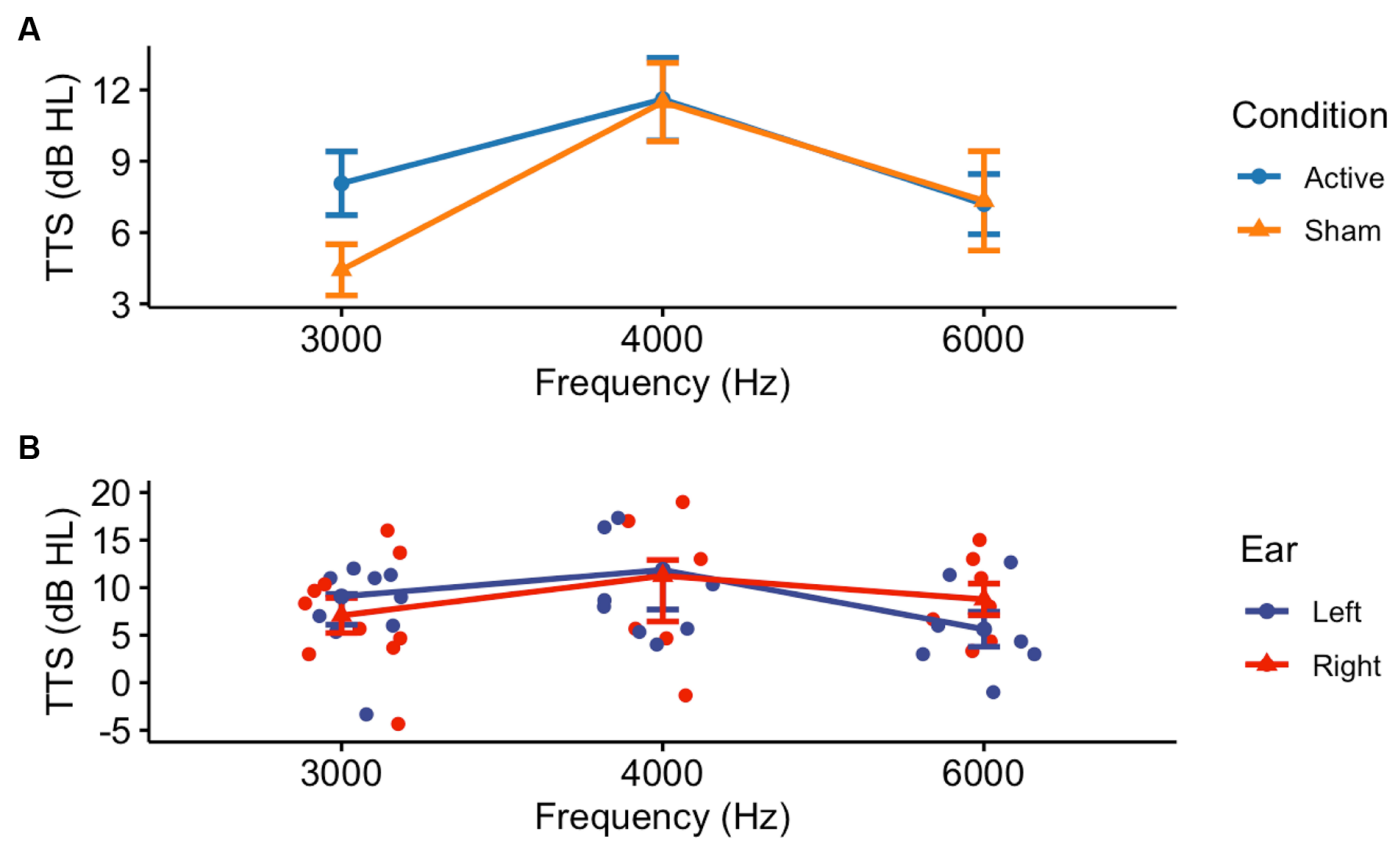
Threshold shifts were observed in the experimental (Active and Sham) groups following noise exposure, though there were no by-ear differences. After receiving pre-noise Active or Sham NIR therapy, participants were subjected to 94 dBA pink noise for 15 min, then underwent post-noise testing including repeat CPATB measures. **(A)** We observed a significant difference between Active and Sham groups at 3000 Hz, but not at 4000 or 6000 Hz (*p* = 0.04, *p* = 0.72, *p* = 0.98, respectively). **(B)** There was no statistical significance between ears at 3000, 4000, or 6000 Hz in the Active group (*p* = 0.54, *p* = 1.00, *p* = 0.18, respectively).

Thresholds shifts following pre-noise treatment and noise exposure in Visits 2 or 4 demonstrated a difference between the Active and Sham groups only at 3000 Hz (Welch Two Sample *t*-test: *t* = 0.212, df = 35, *p* = 0.04) (Figure 3A). There were no differences between Active and Sham conditions when assessing by-ear differences in threshold shifts (Welch Two Sample *t*-test, left ear: *t* = 0.98, df = 47, *p* = 0.33; right ear: *t* = 0.54, df = 42, *p* = 0.59) (Figure 3B). Similarly, when averaged across frequencies, a Kruskal-Wallis test established that there were no significant differences in TTS observed between the Active (Mean ± SD: 9.01 ± 6.29) and Sham (Mean ± SD: 7.55 ± 6.59) devices (*X*^2^ = 0.21, df = 1, *p* = 0.65).

Interestingly, our age subset analysis demonstrated that the TTS observed following both Active and Sham therapy for young adults was statistically different when comparing mean TTS between at least two of the frequencies tested, determined by a one-way ANOVA (Active: *F* = 4.21, df = 2, *p* = 0.03; Sham: *F* = 4.31, df = 2, *p* = 0.02). Tukey’s multiple comparisons for both Active and Sham groups identified the mean TTS at 4000 Hz was larger than that observed at 3000 Hz (Active: *p* = 0.02, 95% CI = [1.00, 13.75]; Sham: *p* = 0.03, 95% CI = [0.66, 12.64]). No significant findings were found in the middle-aged Active or Sham NIR groups (Supplementary Table S2). Overall, the expected protective effect for NIR treatment in lowering TTS as compared to Sham was not observed.

We fitted a linear model (estimated using OLS) to predict TTS with age. The model explained a statistically significant and weak proportion of variance, R2 = 0.04, *F*(1, 118) = 4.54, *p* = 0.035, adj. R2 = 0.03. The model’s intercept, corresponding to Age = 0, is at 13.38 (95% CI [8.22, 18.54], t(118) = 5.14, *p* < 0.001). Within this model, the effect of age was statistically significant and negative, beta = −0.18, 95% CI [−0.35, −0.01], t(118) = −2.13, *p* = 0.035.

### CPATB assessments were not affected by safe, allowable noise exposures

Baseline CPATB and post-noise conditions were conducted in order to assess noise-induced changes in central processing. When examining the differences between pre-noise and post-noise values for all three tests, there were no significant differences observed between the Active and Sham groups. For the control (no noise) active NIR group, there was no difference in CPATB test results between pre- and post-therapy conditions (Supplementary Table S3). For the MRT, there was no significant difference across any SNR schema when compared pre- and post-noise, with the exception of the condition wherein the signal was administered at 78 dB SPL at a SNR of −4 (Wilcoxon rank sum, W = 212, *p* = 0.01) (Table 3).

**Table 3:** Central and peripheral auditory assessment results for pre- and post-noise NIR or sham treatment conditions.

Test	Stage		Active NIR therapy [Mean (SD)]	Sham NIR therapy [Mean (SD)]
Masking level difference test	Pre-noise	*SoNo*	−11.00 (4.88)	−11.65 (3.02)
		*SpiNo*	−19.00 (4.88)	−19.65 (3.02)
		MLD (%correct)	95.46 (7.77)	98.86 (3.11)
	Post-noise	*SoNo*	−11.14 (4.75)	−11.29 (2.64)
		*SpiNo*	−19.14 (4.75)	−19.29 (2.64)
		MLD (%correct)	95.11 (7.06)	97.86 (3.98)
Modified rhyme test	Pre-noise	78 dB, −4 SNR*	77.20 (8.22)	76.27 (7.95)
		78 dB, +4 SNR	91.52 (6.85)	90.91 (5.26)
		70 dB, −4 SNR	76.00 (9.50)	76.46 (9.26)
		70 dB, +4 SNR	93.55 (5.33)	92.82 (5.43)
		70 dB, no noise	97.20 (4.77)	97.48 (3.93)
		% correct	84.90 (4.80)	84.44 (4.07)
	Post-noise	78 dB, −4 SNR	79.00 (8.74)	82.64 (6.00)
		78 dB, +4 SNR	92.73 (5.57)	92.35 (5.66)
		70 dB, −4 SNR	78.00 (8.64)	78.27 (6.18)
		70 dB, +4 SNR	93.73 (4.93)	93.47 (4.97)
		70 dB, no noise	97.90 (3.76)	96.92 (4.08)
		% correct	86.19 (4.10)	86.95 (3.43)
Fixed level frequency test	Pre-noise		16919.14 (1246.26)	17171.05 (1220.39)
	Post-noise		16943.53 (1114.14)	17073.74 (1402.40)

To assess potential therapeutic effects of pre-noise NIR on central auditory processes, Active and Sham NIR post-noise CPATB results were compared. We observed no other significant findings. *denotes a statistically significant difference, *p* < 0.05.

Additionally, the MLD overall percent correct was not statistically different pre- and post-noise (Wilcoxon rank sum test: W = 412.5, *p =* 0.47), suggestive of healthy sensorineural structures resilient to transient but moderate levels of noise. Using Wilcoxon rank sum test, we also found no significant difference in overall MLD values between Active and Sham participants, nor did we observe a difference in mean threshold value (dB S/N) between pre- and post-noise SoNo [−11 (SD ± 4.88); −11.14 (SD ± 4.78)] (W = 485.5, *p* = 0.95) or SπNo [−19 (SD ± 4.88); −19.14 (SD ± 4.75)] conditions (W = 412.5, *p* = 0.45). The mean masking level difference for both Active and Sham groups was 8 ± 0, within well-described normative values. Lastly, there was no significant difference between ears or in pre- and post-noise thresholds during the FLFT. For Active participants, the mean pre- and post-noise FLFT thresholds were 16919.14 (SD ± 1246.26) Hz and 16943.53 (SD ± 1114.14) Hz. On the other hand, Sham participants had mean pre- and post-noise FLFT thresholds of 17171.05 (SD ± 1220.39) Hz and 17073.74 (SD ± 1402.39) Hz, respectively.

## Discussion

This study sought to investigate the effect of OSHA compliant, TTS-inducing noise on auditory health and assess whether pre-noise NIR light therapy can mitigate the effects of noise exposure in healthy human subjects. In general, there are limited clinical studies involving the administration of noise for the examination of prospective therapeutics. Those that have been conducted are variable in their approach, particularly in regard to intensity, the frequency profile, and route of noise administration (i.e., open field versus earphones) (25–28). It is understood that when clinically studying NIHL, TTS models are not only more practical but also ensure increased subject safety in preventing development of permanent threshold shifts (PTS) (13). Furthermore, from a therapeutic perspective, a prophylactic approach is suitable for TTS trials in the context of a pilot study, and could demonstrate therapeutic potential for PTS applications as well (27). To our knowledge, this is the first investigation examining the efficacy of an open-ear 94 dBA pink noise protocol capable of inducing temporary threshold shift without evidence of central or peripheral auditory deficits. The need for a safe and methodical approach to studying NIHL therapeutics remains a priority to bridge the gap between pre-clinical and clinical trials investigating therapeutics for human use, as retrospective studies are generally limited by self-report and animal studies cannot fully recapitulate the complexity of the human auditory system.

In the experimental group, auditory assessments confirmed TTS after noise exposure at all frequencies tested with a full recovery to pre-noise thresholds within 60 min after exposure. We observed larger threshold shifts at 4000 Hz, as compared to 3000 and 6000 Hz, suggestive of a potential tonotopic effect of the noise delivery paradigm utilized herein. This phenomenon has been observed in other studies, wherein threshold shifts were more commonly observed at the high frequencies, including, but not limited to, 4000 Hz (13, 26, 27), though notably the noise intensity and short duration of exposure were not expected to induce high levels of TTS. Additionally, it is important to acknowledge that given the inclusion/exclusion criteria, it potentially allowed people with an unknown degree of existing damage to the cochlea to participate, resulting in a cohort that responded more readily to TTS inducing noise. This in turn may have limited the potential effectiveness of the NIR treatment. Furthermore, in our exploratory analysis we found that middle-aged adults may exhibit increased sensitivity to sound at the high frequencies (including 3000 Hz) or may suffer from TTS more readily compared to younger adults due to additional years of acquired hair cell damage secondary to pre-existing noise exposure, genetics, and age. While we are aware of the limitations of our sample size, these preliminary results were reported to guide future work in this area, as age-related changes to hair cell morphology may potentially play a role in differential hearing profiles following acoustic overexposure. Importantly, no peripheral or central auditory processes were significantly impacted as determined by all functional and perception testing, which were collected both immediately following noise exposure and during all subsequent visits prior to repeated exposures.

In young adults, we observed a possible protective effect of NIR therapy at 4000 Hz, which was supported by the median TTS observed between the Active and Sham devices in that group, though no other group means were significantly different following hypothesis testing. Paradoxically, the observed TTS at 3000 Hz in the Active NIR light therapy group was significantly higher than that observed in the Sham group. We theorize that, along with the small sample size, test–retest variability in the 3000 Hz response is partially responsible for this finding. Conventional and Bekesy audiometric threshold values may vary 5 to 10 dB HL during repeat testing, which may account for the 3.61 dB HL mean difference we observed (24). Indeed, it should be noted that the mean differences between Active and Sham groups fall within this reliability range at all frequencies tested (Table 2). To this end, it is worth noting that elevated levels of distress and reduced concentration following noisy exposures has shown to affect performance in perceptual tasks which could attempt to explain these subtle differences (29). Additionally, it has been shown that adaptations to sound perception can occur following high-level acoustic exposure lessening one’s sensitivity, and therefore rendering low-level sounds less detectable following noise (i.e., when post-noise testing was conducted). Perhaps, the NIR in fact increased the responsiveness of the ear to high-level sound and thereby increased this adaptation effect, therefore explaining this unexpected shift at 3000 Hz. Regardless, it is essential to acknowledge that this therapeutic remains in the early stages of development and these findings need further validation for noise protection regimens in humans.

Notably, our noise protocol provided a faster threshold recovery compared to previous research, where threshold recuperation to baseline required many hours following noise exposure (25, 27). Because TTS recovery is highly dependent on exposure parameters (2), including intensity and duration, we believe that the protocol described herein fills an essential gap in NIHL therapeutic exploration by producing TTS that safely returns to baseline within 1 h of exposure with no indication of prolonged repercussions (i.e., permanent hearing loss, tinnitus). These findings broadly support the novelty and accessibility of our noise protocol and fills an essential platform to understand how noisy environments affect human auditory health.

The inherent challenges of interpreting pilot studies, particularly the small sample size, limit the scope of this work. The authors acknowledge that the observations reported herein, in particular those related to age and sex, should be interpreted with caution and have included them here to reflect their importance in the design of future studies. Notably, there was a low number of female participants due to insufficient threshold shift and subsequent exclusion following the Visit 1 noise exposure, likely consistent with the otoprotective effect of estrogen which has been previously well-described (30, 31). Though no peripheral or central auditory processes were observed to be significantly impacted over the course of this study, we did not explore synaptopathy, nor did we track participants long term for repeat central and auditory processing tests. Noise-induced cochlear synaptopathy in humans remains a controversial topic (32), exacerbated by the limitations of current audiologic diagnostics and is outside the scope of this work. Moreover, while the present prototype theoretically reached the target end organ (33, 34) and cochlear substrate, more work is needed to determine the most efficacious NIR dosing regimen. Additionally, more work is needed to elucidate a therapeutic NIR dosing and delivery protocol in humans and to clarify the pathways involved in otoprotection. Future work should also consider examining gene and protein expression to elucidate how these factors, in conjunction with cell resilience, contribute to the success of NIR therapy as a therapeutic for NIHL.

Although even occupationally allowable levels of noise exposure can induce TTS, these changes are transient in nature and may indicate healthy sensorineural inner ear systems. Locally administered NIR therapy prior to noise did not demonstrate complete protection against noise-induced TTS entirely but may lessen the magnitude of the shift.

## Data availability statement

The original contributions presented in the study are included in the article/Supplementary material, further inquiries can be directed to the corresponding author.

## Ethics statement

The studies involving humans were approved by University of Miami Miller School of Medicine Institutional Review Board. The studies were conducted in accordance with the local legislation and institutional requirements. The participants provided their written informed consent to participate in this study.

## Author contributions

EW: Conceptualization, Data curation, Formal analysis, Investigation, Writing – original draft, Writing – review & editing, Visualization, Project administration. KM: Data curation, Formal analysis, Visualization, Writing – original draft, Writing – review & editing. HG: Conceptualization, Data curation, Supervision, Writing – review & editing, Formal analysis, Investigation. JS: Conceptualization, Writing – review & editing, Investigation. NB: Writing – review & editing, Conceptualization, Investigation, Supervision. NJ: Data curation, Formal analysis, Writing – review & editing. VS: Investigation, Project administration, Writing – review & editing. JC: Investigation, Project administration, Writing – review & editing. SR: Supervision, Writing – review & editing. KY: Conceptualization, Resources, Writing – review & editing. RR: Conceptualization, Resources, Writing – review & editing. MH: Conceptualization, Resources, Supervision, Writing – review & editing.
