# Supplementary material for: Examining the utility of near infrared light as pre-exposure therapy to mitigate temporary noise-induced hearing loss in humans
Source: Front Neurol. 2024 Apr 22;15:1366239. doi: 10.3389/fneur.2024.1366239 (PMC11072974; doi:10.3389/fneur.2024.1366239)
Supplement: Supplementary file 1 [file Table_1.docx]

Supplementary Material

Examining the Utility of Near Infrared Light as Pre-Exposure Therapy to Mitigate Temporary Noise-Induced Hearing Loss in Humans

Erin Williams^1,2,3†^, Kayla Minesinger^1,3†^, Hilary Gallagher^4^, J.R. Stefanson^5^, Nathaniel Bridges^4^, Natalie Jackson^6^, Valerie Stark^7^, Jennifer Coto^1^, Suhrud M. Rajguru^1,3^, Kurt Yankaskas^8^, Rick Rogers^8^, and Michael Hoffer^1,2*^

*** Correspondence:** Michael Hoffer, MD [michael.hoffer@miami.edu](mailto:michael.hoffer@miami.edu)

# Supplementary Figures and Tables

**Table S1. Testing Battery Durations Breakdown.** The qualifying hearing test and screening DPAOEs at the beginning of Visits 1/3 took up to 20 minutes. The subsequent pre-noise testing time approximations are reported and the post-noise testing including 30 minutes of NIR light therapy (Active or Sham) was estimated as well.

|  | **Test** | **Timing** | **Total** |
| --- | --- | --- | --- |
| ***Pre-Noise Testing*** | Abbreviated Hearing Test | ~ 5 minutes | *45 minutes* |
|  | Central Auditory Processing Battery | ~ 15 minutes |  |
|  | Abbreviated hearing test repeat | ~ 5 minutes |  |
| ***Post-Noise Testing*** | Abbreviated Hearing Test | ~5 minutes | *60 minutes* |
|  | Central Auditory Processing Battery | ~15 minutes |  |
|  | *NIR Light therapy (Active or Sham)* | *30 minutes* |  |
|  | DPOAEs | ~5 minutes |  |
|  | Recovery Hearing test | ~5 minutes |  |

**Table S2.** **Age-Specific TTS Results for NIR or Sham Treatment Conditions**. To determine potential age-related effects of pre-noise NIR on TTS, Active and Sham NIR mean TTS results were compared in an exploratory analysis. We observed no significant differences between Sham and Active age-specific groups.

| *Condition* | | *FREQUENCY TESTED* | | |
| --- | --- | --- | --- | --- |
|  |  | **3000 Hz (Mean (SD))** | **4000 Hz (Mean (SD))** | **6000 Hz**  **(Mean (SD))** |
| Young | *Active NIR Therapy* | 6.50 (4.57) | 13.88 (6.86) | 8.94 (3.99) |
|  | *Sham NIR Therapy* | 5.17 (4.76) | 11.82 (6.55) | 5.46 (7.26) |
| Middle-Age | *Active NIR Therapy* | 6.28 (6.27) | 5.33 (4.07) | 3.44 (2.53) |
|  | *Sham NIR Therapy* | 1.83 (3.01) | 7.22 (1.50) | 5.67 (1.41) |

| **Test** | **Stage** | | **Active NIR Therapy (Mean [SD])** | **Sham NIR Therapy (Mean [SD])** |
| --- | --- | --- | --- | --- |
| Masking Level Difference Test | Pre-Noise | *SoNo*  *SpiNo*  MLD (%Correct) | -11.00 (4.88) | -11.65 (3.02) |
|  |  |  | -19.00 (4.88) | -19.65 (3.02) |
|  |  |  | 95.46 (7.77) | 98.86 (3.11) |
|  | Post-Noise | *SoNo*  *SpiNo*  MLD (%Correct) | -11.14 (4.75) | -11.29 (2.64) |
|  |  |  | -19.14 (4.75) | -19.29 (2.64) |
|  |  |  | 95.11 (7.06) | 97.86 (3.98) |
| Modified Rhyme Test | Pre-Noise | 78 dB, -4 SNR  78 dB, +4 SNR  70 dB, -4 SNR  70 dB, +4 SNR  70 dB, no noise  % Correct | 77.20 (8.22) | 76.27 (7.95) |
|  |  |  | 91.52 (6.85) | 90.91 (5.26) |
|  |  |  | 76.00 (9.50) | 76.46 (9.26) |
|  |  |  | 93.55 (5.33) | 92.82 (5.44) |
|  |  |  | 97.20 (4.77) | 97.48 (3.93) |
|  |  |  | 84.90 (4.80) | 84.44 (4.07) |
|  | Post-Noise | 78 dB, -4 SNR  78 dB, +4 SNR  70 dB, -4 SNR  70 dB, +4 SNR  70 dB, no noise  % Correct | 79.00 (8.74) | 82.64 (5.99) |
|  |  |  | 92.73 (5.57) | 92.35 (5.66) |
|  |  |  | 78.00 (8.64) | 78.27 (6.18) |
|  |  |  | 93.73 (4.93) | 94.47 (4.97) |
|  |  |  | 97.90 (3.76) | 96.92 (4.08) |
|  |  |  | 86.19 (4.10) | 86.95 (3.43) |
| Fixed Level Frequency Test | Pre-Noise | | 16919.14 (1246.26) | 17171.05 (1220.39) |
|  | Post-Noise | | 16943.53 (1114.14) | 17073.74 (1402.39) |

**Table S3.** **Central and Peripheral Auditory Assessment Results for Control Pre- and Post-Quiet Period NIR or Sham Treatment Conditions**. To assess effects of pre-noise NIR on central auditory processes, Active and Sham NIR post-quiet period CPATB results were compared. We observed no significant differences between pre- and post-quiet groups.
